# Profile of miRNAs induced during sheep fat tail development and roles of four key miRNAs in proliferation and differentiation of sheep preadipocytes

**DOI:** 10.3389/fvets.2024.1491160

**Published:** 2024-12-03

**Authors:** Wei Zhang, Shiyin Wang, Liwei Yang, Li Gao, Chengcheng Ning, Mengsi Xu, Shuangyi Deng, Shangquan Gan

**Affiliations:** ^1^Key Laboratory of Livestock and Poultry Healthy Breeding Technology in Northwest China, Xinjiang Agricultural Vocational and Technical University, Changji, China; ^2^State Key Laboratory of Sheep Genetic Improvement and Healthy Production, Xinjiang Academy of Agricultural and Reclamation Sciences, Shihezi, China; ^3^College of Coastal Agricultural Sciences, Guangdong Ocean University, Zhanjiang, China

**Keywords:** miRNA, sheep, fat tissue, tail, preadipocyte, adipogenesis

## Abstract

**Background:**

The fat tail of sheep is an adaptive trait that facilitates their adaptation to harsh natural environments. MicroRNAs (miRNAs) have been demonstrated to play crucial roles in the regulation of tail fat deposition.

**Methods:**

In this study, miRNA-Seq was employed to investigate the expression profiles of miRNAs during different developmental stages of sheep fat tails and elucidate the functions of differentially expressed miRNAs (DE miRNAs).

**Results:**

A total of 350 DE miRNAs were identified, among which 191, 60, 26, and 21 were significantly upregulated in tail fat tissues of fetal, lamb, hogget Altay sheep, and adult Xinjiang fine wool (XFW) sheep but downregulated in other stages. Furthermore, we predicted a set of candidate target genes (4,476) for the top 20 DE miRNAs. Gene ontology (GO) and Kyoto Encyclopedia of Genes and Genomes (KEGG) analysis showed that they involve in several adipogenesis-related pathways. Subsequent investigations indicated that four DE miRNAs, miR-433-3p, miR-485-3p, miR-409-3p, and miR-495-3p, could suppress the expression of peroxisome proliferator-activated receptor gamma (*PPARγ*) and phosphoinositide-3-kinase regulatory subunit 3 (*PIK3R3*) and regulate the preadipocyte development in sheep. Meanwhile, the lipid metabolism-related genes, fatty acid-binding protein (*FABP3*), perilipin 1 (*PLIN1*), adiponectin C1Q and collagen domain containing (*ADIPOQ*), and lipoprotein lipase (*LPL*), were significantly downregulated (*p* < 0.01).

**Conclusion:**

The expression patterns of miRNAs exhibited significant fluctuations during different development periods of the fat tail, and some of them may participate in the regulation of tail fat deposition by modulating the proliferation and differentiation of preadipocytes.

## 1 Introduction

Thin-tailed sheep (*Ovis aries*) were domesticated in the Neolithic period about 11,000 years ago (1, 2). Approximately 5,000 years ago, a subset of thin-tailed sheep breeds underwent evolutionary changes through prolonged artificial and natural selection processes, resulting in the emergence of fat-tailed ones (3–5). Presently, approximately 25% of sheep breeds are fat-tailed, and most of them are distributed in temperate and cold regions of the earth (6). In China specifically, indigenous fat-tailed sheep account for 80% of all local breeds. The main reason for this phenomenon is that these areas have a longer season when grass is scarce and a relatively shorter season when the grass is abundant. Fat-tailed sheep can store large amounts of fat in their tails during summer and autumn and then obtain energy by decomposing tail fat in cold winter and spring; thus, they are more adapted to the harsh natural environment than thin-tailed ones. In addition, the fat tissues of sheep also serve as an important energy source for human populations residing within these areas (7, 8).

Over the past few years, there has been a significant shift in people’s attitudes toward meat consumption. Given that the fatty meat consumption greatly increases the risk of cardiovascular and cerebrovascular diseases, an increasing number of people have chosen to consume low-fat meat. Therefore, excessive fat deposition would substantially diminish the economic value of mutton (9–11). Furthermore, in intensive and semi-intensive sheep industry systems, a balanced supply of nutrients could be guaranteed throughout the year, and sheep do not need to store excess fat in their tails to enhance their adaptability to the environment. Hence, numerous fat-tailed sheep breeds need breeding to reduce the carcass fat content, and elucidating the molecular regulatory mechanism underlying tail fat deposition in sheep will positively affect the breeding of leaner sheep breeds (12, 13).

**Figure 1:**
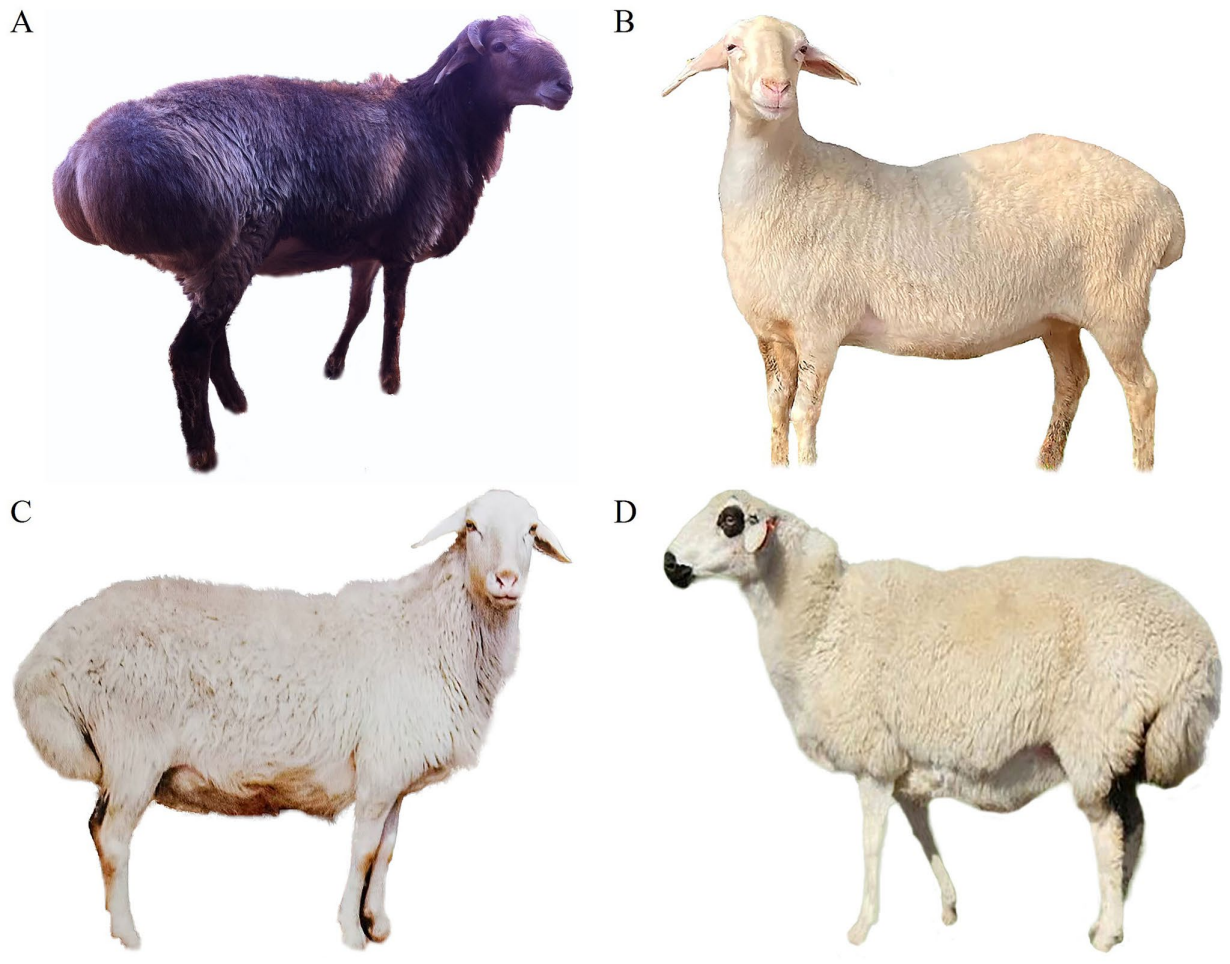
Altay sheep in the present study **(A)** and Hu sheep **(B)**, Guangling large-tailed sheep **(C)**, Sunit sheep **(D)** involved in the prior investigation (16–18).

MicroRNAs (miRNAs) are a class of small non-coding RNA. They bind to target mRNA by the seed regions to exert their effects on gene expression at the posttranscriptional level (14). In recent years, the differentially expressed miRNAs (DE miRNAs) in tail fat tissue of fat-tailed and thin-tailed sheep were investigated using high-throughput sequencing, and plenty of miRNAs that play crucial roles in sheep tail fat deposition were identified (15–18). Altay sheep inhabit northwest China (Figure 1), characterized by long and cold winters with an average annual temperature ranging from 0.7°C to 4.9°C and extreme minimum temperatures as low as −51.5°C can be experienced. Hence, Altay sheep possess a remarkable ability for fat deposition. Their tails and rumps are fused together, and the rump fat weight of adult male sheep accounted for approximately ~25% of carcass weight on average during autumn (19). Therefore, Altay sheep could adapt proficiently to the local harsh natural environment. In comparison with the fat-tailed sheep involved in previous investigations, Altay sheep exhibit a stronger tail fat deposition ability and more distinct fat-tail traits. Hence, it is a more ideal model for studying tail fat deposition.

Here, we collected tail fat tissues from four different developmental stages of fat-rumped Altay sheep and adult thin-tailed Xinjiang fine wool sheep (XFW). We applied miRNA-Seq to detect DE miRNAs and then further investigated the functions of key miRNAs and their targets in fat deposition. Our results will provide a solid theoretical basis for the breeding improvement of fat-tailed sheep.

## 2 Materials and methods

### 2.1 Tissue collection and RNA extraction

To investigate the profile of miRNAs induced during sheep fat tail development, tail fat tissues of four key developmental stages of fat-rumped Altay sheep were collected. Tail fat tissues of adult thin-tailed XFW sheep were collected simultaneously to detect the DE miRNAs between adult Altay and XFW sheep.

Six pregnant adult Altay ewes with similar due dates were selected. Three of them were slaughtered on the 120th day of gestation to collect the tail fat tissues of fetuses. The rest of the three ewes were fed until they gave birth, and the tail fat of the 1-month-old lambs were collected. Meanwhile, three tail fat tissues of 1- and 2-year-old Altay ewes as well as 2-year-old XFW ewes were collected, respectively. All sheep were supplied and uniformly fed by the farm of Xinjiang Academy of Agricultural and Reclamation Sciences. Prior to sampling, the sheep were anesthetized by injecting 30 mg/kg body weight of pentobarbital sodium (Ningbo, Zhejiang, China) via the ear vein (20). Pentobarbital sodium is a type of medium-efficiency barbital hypnotics that can inhibit the uplink activation system of the brain stem reticular structure. Approximately 50 g of tail fat tissue from each sheep was rapidly collected and immersed in liquid nitrogen for transportation and then stored at −80°C in the laboratory. Tail fat tissues of three sheep were collected at each stage, with a total of 15 samples collected, including fetuses, 1-month-old lambs, 1- and 2-year-old Altay ewes, and 2-year-old XFW ewes, respectively.

Total RNA was isolated from tissues using TRIzol reagent (Invitrogen, United States) according to the manufacturer’s protocol. The concentration and integrity of the total RNA were assessed using 1% agarose gel electrophoresis and 2,100 Bioanalyzer Instrument (Agilent Technologies, CA, United States) by measuring the absorbance at 260 and 280 nm.

### 2.2 MiRNA library construction and sequencing

The concentration of three RNA samples from each developmental stage was standardized and then mixed into one sample to prepare for the miRNA sequencing library using TruSeq Small RNA Sample Prep Kits (Illumina, San Diego, CA, United States). Subsequently, five distinct libraries were constructed and designated as fetal fat rump, lamb fat rump, hogget fat rump, adult fat rump, and adult thin tail, respectively. All miRNA libraries underwent single-end sequencing on an Illumina HiSeq 2,500 at BGI (Shenzhen, China).

### 2.3 Sequencing data analysis and identification of miRNA

The raw data were processed using software developed by BGI (Shenzhen, China) to get clean reads following the criteria outlined below: (1) low-quality reads (more than 10% bases could not be determined); (2) reads with 5′ primer contaminants; (3) reads without 3′ primer; (4) reads without the insert tag; (5) reads with poly A; and (6) reads shorter than 18 nt. Subsequently, the reads with a sequence length ranging from 18 to 26 nt were retained, and the reads including common RNA families (rRNA, tRNA, snRNA, and snoRNA) and repeats were eliminated using Ensembl, RFam, and Repbase databases. Thereafter, the length distribution of these filtered valid reads was summarized, including the summary of unique tags and total tags. The small RNAs with unique sequences ranging from 18 to 26 nt were aligned to the miRNA precursor of *Ovis aries* and *Bos taurus* in miRbase 21.0 (accessed on 20 February 2021)^1^ to obtain the miRNA count as well as the base bias on the first position of the identified miRNAs with certain length and at each position of all identified miRNAs, respectively. The locations of mapped miRNAs were further determined by aligning against the sheep genome Oar_v3.1 (accessed on 20 February 2021).^2^ The unmapped unique sequences were utilized to predict novel miRNAs applying Mireap software (BGI, Shenzhen, China) by analysis of the secondary structure, identification of the Dicer cleavage site, and determination of the minimum free energy.

### 2.4 Differential expression analysis

The miRNA expression levels in two libraries (control and treatment) were normalized to obtain the expression of transcript per million (TPM), calculate fold change and *p*-value from the normalized expression using DESeq2 (21). The miRNAs satisfied the following conditions: log2 (fold change) ≥ 1 or ≤ −1 and *p* < 0.05 were identified as significant DE miRNAs.

### 2.5 qRT-PCR validation

The reliability of RNA-seq data was validated by randomly selecting six upregulated miRNAs in fetal fat rump and six downregulated ones in hogget fat rump using stem-loop real-time quantitative PCR. The expression levels of each miRNA were calculated using the 2^-∆∆CT^ method, with the expressions of 5S serving as a reference for relative quantification. The primer information is shown in Supplementary Table S1.

### 2.6 Target genes prediction and functional analysis of DE miRNAs

TargetScan^3^ and miRanda^4^ were utilized to predict the target genes of DE miRNAs. The predicted genes, for which TargetScan Total context score < −0.2, *P*_CT_ > 0.5, and miRanda energy < −20, were considered as candidate target genes. Then, the target genes were annotated by Gene ontology (GO)^5^ and enriched using the Kyoto Encyclopedia of Genes and Genomes (KEGG) biological pathway database,^6^ respectively. The *p*-value was corrected using the Bonferroni method, and *p* ≤ 0.05 was considered as significantly enriched terms (22).

### 2.7 Dual-luciferase reporter assays

Four DE miRNAs, namely oar-miR-433-3p, oar-miR-485-3p, oar-miR-409-3p, and oar-miR-495-3p, which were significantly expressed in the tail fat tissues at different developmental stages of Altay sheep, and their candidate target genes, *PPARγ*, *PDCD4*, and *PIK3R3*, which were enriched in pathways related to fat deposition, were selected for further verification of their target relationship using dual-luciferase reporter assays. The prediction results of target genes indicated that there is one binding site of oar-miR-433-3p in the 3’UTR of *PPARγ* mRNA, one binding site of oar-miR-485-3p in the 3’UTR of *PDCD4* mRNA, and three binding sites of oar-miR-485-3p, oar-miR-409-3p, and oar-miR-495-3p in the 3’UTR of *PIK3R3* mRNA, respectively.

The wild-type 3’UTR of the *PPARγ*, *PDCD4*, and *PIK3R3* mRNA was amplified and inserted into the psiCHECH2 vector (Promega, Madison, WI, United States) between the *Xho*I and *Not*I restriction enzyme cutting sites and named psiCHECH2-*PPARγ*-3’UTR-WT, psiCHECH2-*PDCD4*-3’UTR-WT, psiCHECH2-*PIK3R3*-3’UTR-WTI, psiCHECH2-*PIK*3*R*3-3’UTR-WTII, and psiCHECH2-*PIK3R3*-3’UTR-WTIII, respectively. Their mutant type 3’UTR was generated using the Site-Directed Mutagenesis Kit (Thermo Fisher Scientific, MA, United States) and named psiCHECH2-*PPARγ*-3’UTR-Mut, psiCHECH2-*PDCD4*-3’UTR-Mut, psiCHECH2-*PIK3R3*-3’UTR-MutI, psiCHECH2-*PIK3R3*-3’UTR-MutII, and psiCHECH2-*PIK3R3*-3’UTR-MutIII, respectively. The primers used in plasmid construction are shown in Supplementary Table S1.

The vectors and their corresponding miRNA mimics (GenePharma, Shanghai, China), which were fluorescently labeled with 6-FAM, along with psiCHECK2 pure vectors with negative control (NC), and psiCHECK2 pure vectors with miRNA mimics were co-transfected into 293 T cells using Lipofectamine 3,000 Transfection Kit (Thermo Fisher Scientific, MA, United States). After 6 h, the culture medium was replaced, and the transfection efficiency was assessed by detecting the fluorescence of 6-FAM under an inverted fluorescence microscope (Olympus, Japan). Then, the cells were cultured for an additional 48 h, and the relative luciferase activity in the cell was measured by Dual-Luciferase Reporter Assay System (Promega, Madison, WI, United States) according to the manufacturer’s protocol. Each treatment group underwent three replicates.

### 2.8 Transfection of miRNAs into sheep preadipocytes

The sheep preadipocytes obtained through primary culture in our laboratory were seeded in 6-well plates at a density of 1 × 10^5^ cells/well. When the cells reached a confluence of 70%, the following molecules, the 6-FAM fluorescently labeled mimics and negative controls for oar-miR-433-3p, oar-miR-485-3p, oar-miR-409-3p, and oar-miR-495-3p, as well as inhibitors and negative controls for the same miRNAs (GenePharma, Shanghai, China), were transfected into the cells, respectively, using Lipofectamine 3,000 Transfection Kit (Thermo Fisher Scientific, MA, United States) according to the manufacturer’s protocol. The concentrations of miRNA mimics, inhibitors, and NC were maintained at 50 nM within the recommended range provided by the manufacturer. Six hours later, the culture medium was replaced, and total RNA and protein were extracted to detect the expression levels of candidate target genes *PPARγ*, *PDCD4*, and *PIK3R3* when the cells were further cultured for 48 and 72 h.

### 2.9 qRT-PCR analysis

The mRNA and protein levels of candidate target genes were detected by qRT-PCR and Western blot, and the primers are listed in Supplementary Table S1. The qRT-PCR analysis was carried out on a Roche 480 instrument (Roche, Mannheim, Germany) using the SYBR Green PCR Master Mix Kit (QIAGEN, Germany) in accordance with the manufacturer′s instructions. The reaction mixture consisted of 10 μL 2 × Quanti Fast SYBR Green PCR Master Mix, 1 μL cDNA (<100 ng), 0.5 μL forward and reverse primers (10 μM), respectively, and ddH_2_O to a total volume of 20 μL. The following conditions were used for amplification: 95°C for 5 min, followed by 45 cycles of 95°C for 10 s, 60°C for 30 s, and 72°C for 7 min. qRT-PCR analysis was conducted in triplicate for each sample, and relative expression for each gene was estimated using the 2^-△△Ct^ method.

### 2.10 Cell proliferation capacity analysis

The MTT cell proliferation detection kit (Solarbio, Beijing, China) was used to assess the influence of miRNAs on the proliferative capacity of sheep preadipocytes in accordance with the manufacturer’s protocol. The sheep preadipocytes were seeded in 96-well plates at a density of 1 × 10^4^ cells/well. When the cells reached a confluence of 50%, the mimics, inhibitors, and NC of oar-miR-433-3p, oar-miR-485-3p, oar-miR-409-3p, and oar-miR-495-3p were transfected into the cells according to the methods mentioned above. After the cells were further cultured for 72 h, the culture medium was removed carefully, 90 μL of complete culture medium and 10 μL of MTT solution were added per well, cultured in a cell incubator at 37°C and 5% CO_2_ for 4 h, then the culture medium was removed, 110 μL of formazan solution per well was added, and were shaken on a shaker for 10 min at low speed to fully dissolve the crystal. Then, the *OD* values of each well were measured at a wavelength of 490 nm. To eliminate background noise, the blank wells were zeroed by adding 110 μL of formazan solution. Subsequently, the average *OD* values for each treatment were calculated based on six replicates.

### 2.11 Cell-induced adipogenic differentiation capacity analysis

The sheep preadipocytes were seeded in six-well plates at a density of 1 × 10^5^ cells per well. When the cells reached a confluence of 90% ~ 100%, an adipogenic differentiation induction medium was introduced, consisting of 89% DMEM/F12 (Gibco, NY, United States), 10% FBS (Gibco, NY, United States), and 1% penicillin–streptomycin (Solarbio, Beijing, China), supplemented with 80 μmol/L oleic acid (Sigma, MO, United States), 0.25 mmol/L IBMX (Sigma, MO, United States), 5 μmol/L DEX (Sigma, MO, United States), and 8 μmol/L INS (Sigma, MO, United States). Following a 4-day induction period in this medium, the induction medium was replaced with a maintenance medium comprising 89% DMEM/F12, 10% FBS, 1% penicillin–streptomycin, and 5 μmol/L INS for an additional 2 days of culture. Thereafter, the induction and maintenance medium were changed every 2 days until the 12th day (23, 24). Meanwhile, on the 3rd, 6th, and 9th days of induced differentiation, the miRNA mimics, inhibitors, and NC of oar-miR-433-3p, oar-miR-485-3p, oar-miR-409-3p, and oar-miR-495-3p were transfected into the cells to ensure that their levels remained as elevated as possible. Subsequently, the expression levels of four lipid metabolism-related genes, FABP3, PLIN1, ADIPOQ, and LPL, were detected using Western blot.

To evaluate the influence of cellular miRNA levels on their induced lipogenic differentiation ability, the Oil Red O Staining Kit (Solarbio, Beijing, China) was used to compare the extent of lipid accumulation in cells subjected to different treatments on the 12th day of induced differentiation. The staining of Oil Red O was conducted according to the instructions of the kit. After visual observation and photography of stained cells, 1 mL isopropyl alcohol was added to each well for Oil Red O extraction, and the *OD* value at 510 nm was determined. Subsequently, DNA was extracted from the cells after extraction of Oil Red O using phenol–chloroform–isoamyl alcohol extraction method; the amount of DNA was calculated by measuring the *OD* value at 260 nm; and then, the amount of fat deposition in the induced differentiated cells was determined through calculation of Oil Red O *OD*510/DNA *OD*260.

### 2.12 Western blot analysis

The cells were extracted, and 1 mL of pre-cooling RIPA lysis buffer containing 1 mM PMSF was added to obtain the total protein. The total protein concentration was determined using the TaKaRa BCA Protein Assay Kit (TaKaRa, Beijing, China) following the manufacturer’s protocol. The proteins were separated using a 12% SDS-PAGE gel (Solarbio, Beijing, China) at 110 V for 90 min and subsequently transferred onto a PVDF membrane (Millipore, United States) at 400 mA for 50 min. The membrane was sealed at 4°C overnight with blocking buffer (TaKaRa, Beijing, China) and washed three times using TBST (Solarbio, Beijing, China). It was then probed with rabbit polyclonal antibody against PPARγ (1:2,000, Abcam, Cambridge, United Kingdom), PDCD4 (1:2,000, Abcam, Cambridge, United Kingdom), PIK3R3 (1:2000, Abcam, Cambridge, United Kingdom), FABP3 (1:2,000, Proteintech, Wuhan, China), PLIN1 (1:5,000, Proteintech, Wuhan, China), ADIPOQ (1:200, Proteintech, Wuhan, China), LPL (1:1,000, Proteintech, Wuhan, China), and beta-actin (1:2,000, Abcam, Cambridge, United Kingdom) at 4°C overnight. Following this incubation period, the membrane was washed three times for 10 min each. Then, it was incubated for 1 h at room temperature with a goat anti-rabbit IgG secondary antibody (1:5,000, Proteintech, Wuhan, China) labeled with HRP, followed by washing three times, 10 min each time. Finally, the reaction bands were developed using the SuperSignal West Femto Trial Kit (Thermo Fisher Scientific, MA, United States) and detected and analyzed using a chemiluminescence imaging system (Vilber, Paris, France).

### 2.13 Statistical analysis

Statistical analyses were conducted on a minimum of three independent experiments for each treatment, and all data were presented as the means ± standard deviation. All statistical analyses were performed using SPSS version 20 (IBM, NY, United States). Comparisons between two groups were performed by the *t*-test, while comparisons among multiple groups carried out using a one-way analysis of variance followed by Tukey’s multiple comparisons test; *p* < 0.05 and *p* < 0.01 were considered as statistically significant differences.

## 3 Results

### 3.1 Profiles of sequencing data

**Figure 2:**
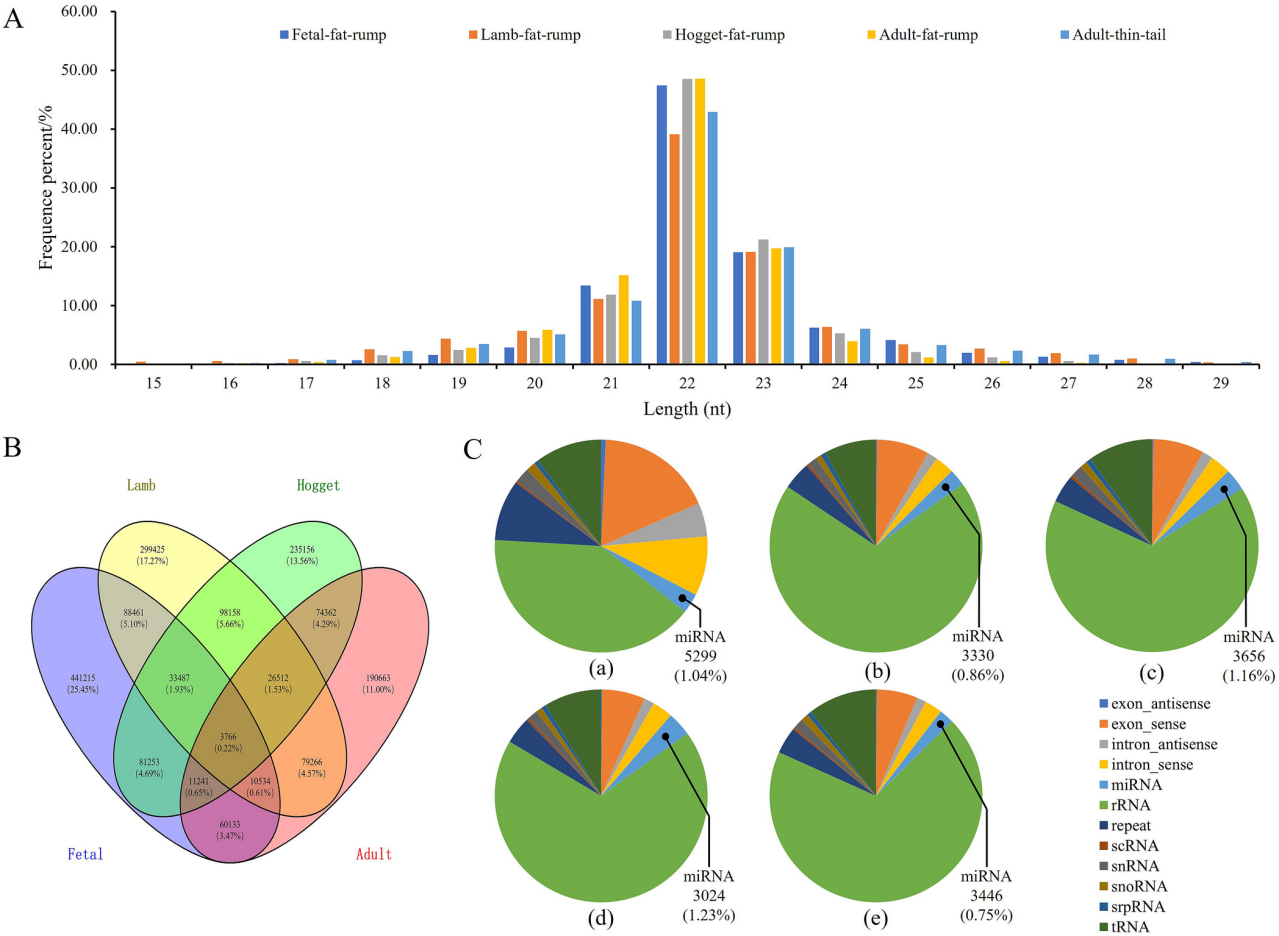
Profiles of sequencing data, **(A)** length distribution of unique sRNAs, **(B)** Venn chart of unique sRNAs expression during four different development stages of Altay sheep, **(C)** constitute of five sRNA libraries.

To explore the miRNA expression profiles in tail fat tissues of sheep during different development stages, five sRNA libraries, namely, fetal fat rump, lamb fat rump, hogget fat rump, adult fat rump, and adult thin tail respectively, were constructed and sequenced. The raw data were filtered to obtain the total sRNAs and unique sRNAs (Supplementary Table S2). More than 98% of the sRNAs ranged from 18 nt to 26 nt in length (Figure 2A). There were 441,215 unique sRNAs expressed exclusively during the fetal stage, 299,425 during the lamb stage, 235,156 during the hogget stage, and 190,663 during the adult stage. Additionally, there were also 3,766 unique sRNAs expressed simultaneously across these four stages (Figure 2B). Then, the unique sRNAs were classified and annotated, and 5,299, 3,330, 3,656, 3,024, and 3,446 miRNAs were identified from tail fat tissue of fetal, lamb, hogget, adult Altay sheep, and adult XFW sheep (Figure 2C).

### 3.2 DE miRNA analysis

**Figure 3:**
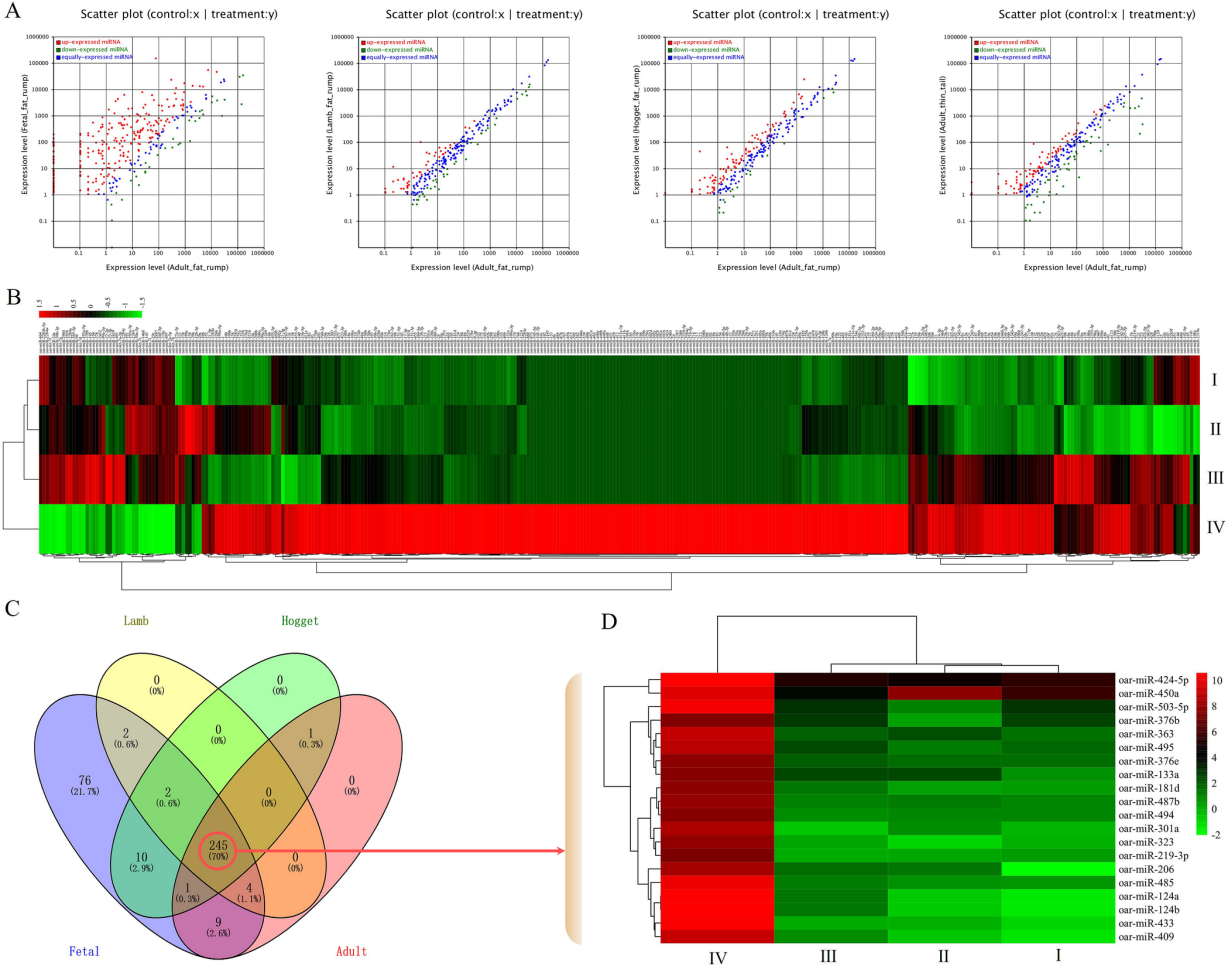
Characterization of DE miRNAs, **(A)** scatter plot of DE miRNAs, **(B)** heatmap of DE miRNAs, **(C)** Venn chart of DE miRNAs during four different developmental stages of Altay sheep, **(D)** heatmap of top 20 DE miRNAs, I, II, III, and IV indicated adult thin tail/adult fat rump, hogget fat rump/adult fat rump, lamb fat rump/adult fat rump, and fetal fat rump/adult fat rump, respectively.

The miRNA expression of adult Altay sheep was used as the control, while fetal, lamb, hogget Altay sheep, and adult XFW sheep were considered as treatment groups to normalize their expression. The fold change and *p*-value were calculated to identify the significant DE miRNAs. A total of 350 DE miRNAs, which log2 (fold change) ≥ 1 or ≤ −1 and *p* < 0.05, were identified (Figure 3A; Supplementary Table S3). The cluster analysis and heatmap drawing were performed using the Heatmap Plot tool in Hiplot Pro^7^ (Figure 3B). Among these DE miRNAs, 191, 60, 26, and 21 miRNAs were extremely upregulated in tail fat tissues of fetal, lamb, hogget Altay sheep, and adult XFW sheep, respectively, but were significantly downregulated in other development stages. Then, their expression profiles across different developmental stages of Altay sheep were further analyzed. From a pool of 245 DE miRNAs that exhibited significant differential expression among these four development stages, the top 20 DE miRNAs were selected for further investigation (Figures 3C,D).

### 3.3 Validation of sequencing data

**Figure 4:**
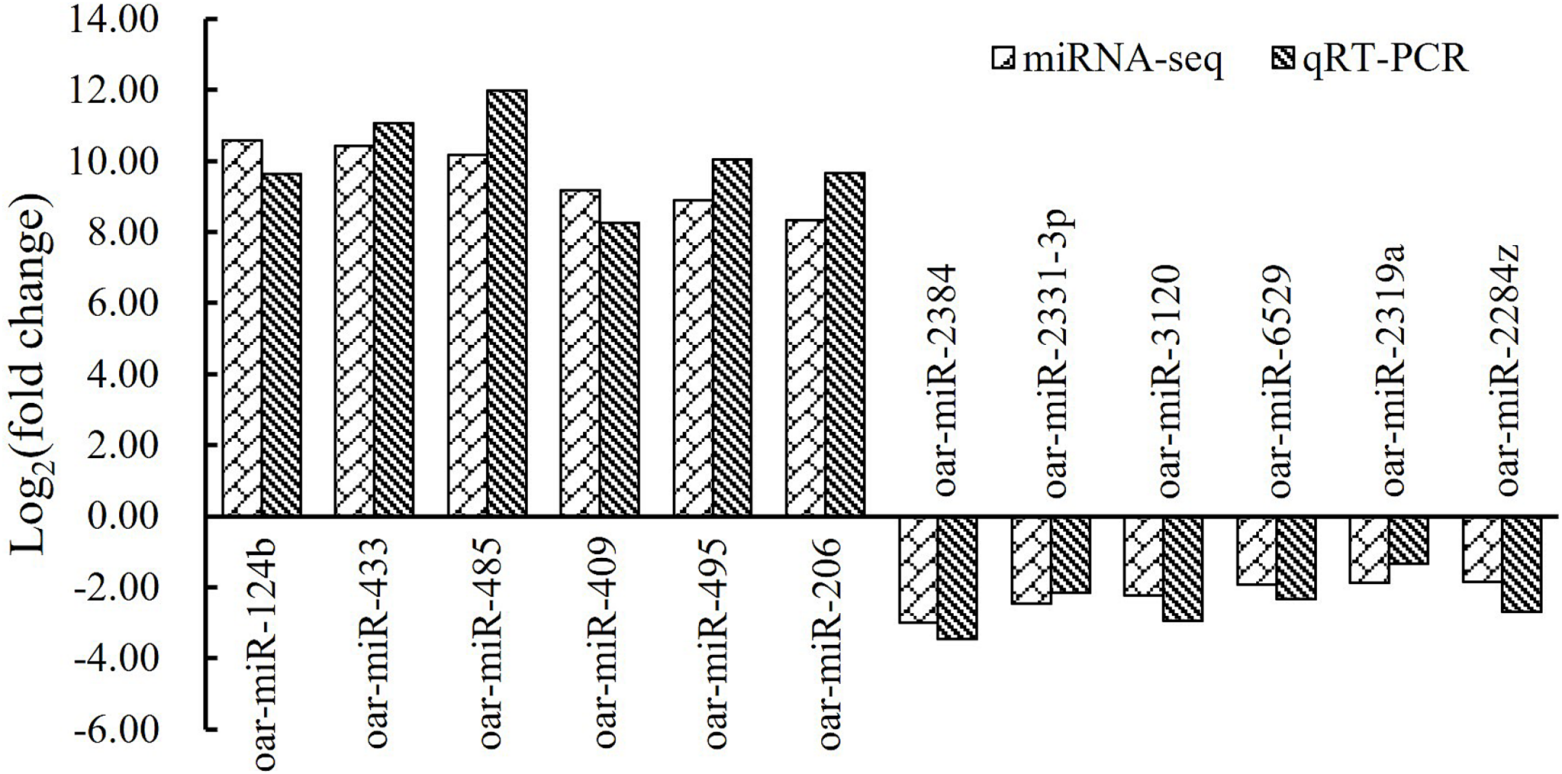
MiRNA sequencing data and qRT-PCR results of six upregulated miRNAs and six downregulated ones.

To validate the sequencing data, a total of six upregulated miRNAs in fetal fat rump and six downregulated ones in hogget fat rump were randomly selected, and their expression levels were detected in tail fat tissues of corresponding developmental stages using stem-loop real-time quantitative PCR. By analyzing the qRT-PCR results and miRNA sequencing data, we found that the fold change of selected miRNAs showed similar trends (Figure 4), indicating that the miRNA sequencing data were reliable and efficient.

### 3.4 Candidate target genes and functional analysis of top 20 DE miRNAs

**Figure 5:**
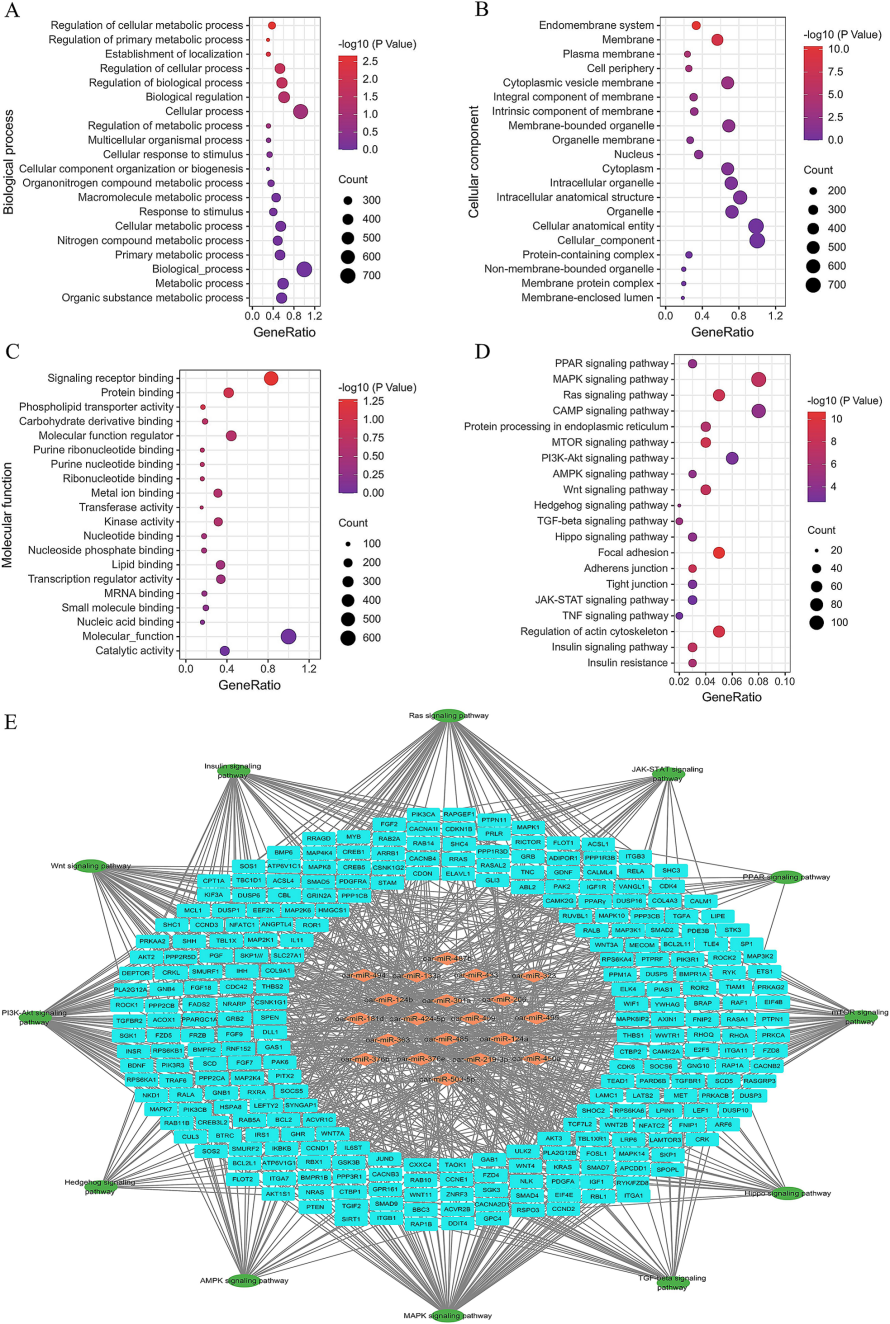
Significantly enriched Gene Ontology and KEGG of the candidate target genes of top 20 DE miRNAs, **(A)** top 20 biological process terms, **(B)** top 20 cellular component terms, **(C)** top 20 molecular function terms, **(D)** top 20 KEGG terms, **(E)** regulatory network formed by miRNAs, candidate target genes, and signaling pathway.

A total of 4,476 candidate target genes of the top 20 DE miRNAs were predicted (Supplementary Table S4), and their biological functions were analyzed by using GO and KEGG enrichment analysis (Supplementary Table S5). Ninety-nine GO terms were significantly enriched, including regulation of cellular metabolic process (GO:0031323), regulation of primary metabolic process (GO:0080090), phospholipid transporter activity (GO:0005548), and lipid binding (GO:0008289) that are associated with fat metabolism. The top 20 GO terms are shown in Figures 5A–C.

The top 20 KEGG terms are presented in Figure 5D, while the regulatory network of miRNAs, candidate target genes, and associated signaling pathways are depicted in Figure 5E. Several pathways, such as the PPAR signaling pathway (ko03320), AMPK signaling pathway (ko04152), insulin signaling pathway (ko04910), mTOR signaling pathway (ko04150), and PI3K-AKT signaling pathway (ko04151), have already been proved playing critical roles in adipogenesis (25). Meanwhile, the interaction analysis revealed that these miRNAs intricately interconnected these signaling pathways through the regulation of their respective target genes (Figure 5E).

Among these top DE miRNAs and candidate genes, the expression of miR-433-3p, miR-485-3p, miR-409-3p, and miR-495-3p was significantly different in the tail fat tissues at different developmental stages of Altay sheep. Their candidate target genes, *PPARγ*, *PDCD4*, and *PIK3R3*, have been shown to play key roles in adipocyte proliferation, differentiation, and lipid deposition (25). Therefore, the regulatory relationship between these four miRNAs and their three candidate genes was further validated, and their functions in adipocyte development and lipid deposition were further investigated.

### 3.5 Target genes validation of key miRNAs

**Figure 6:**
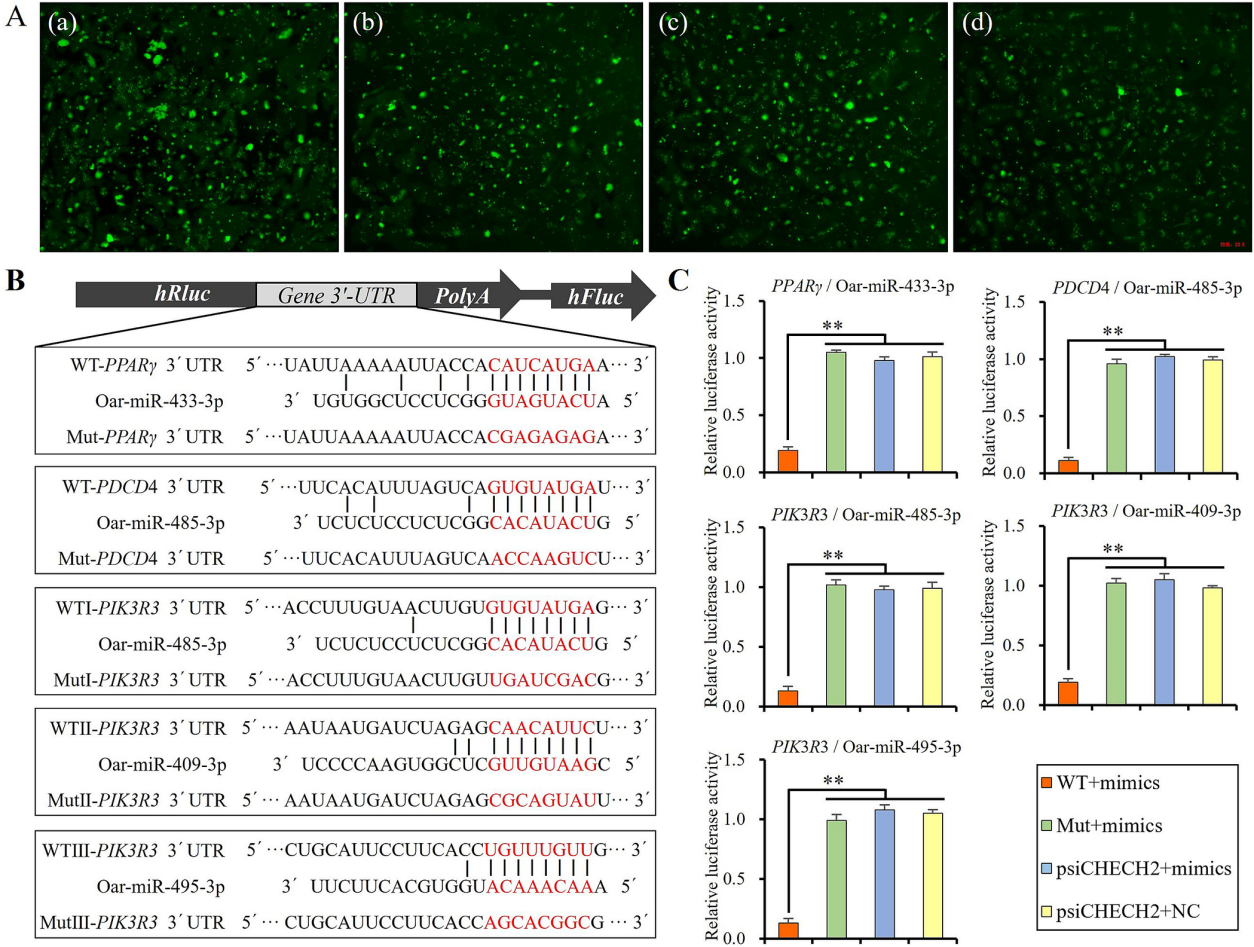
Verifying the targeted regulatory relationship using dual-luciferase assay, **(A)** fluorescence image of 293 T cells 6 h after transfecting plasmid and mimics of miR-433-3p (a), miR-485-3p (b), miR-409-3p (c), and miR-495-3p (d), **(B)** schematic diagram of miRNAs and their target sites, **(C)** relative luciferase activity detecting results; **indicates significant difference (*p* < 0.01).

The miRNA mimics were labeled with 6-FAM for easy evaluation of transfection efficiency by fluorescence detection using an inverted fluorescence microscope. Fluorescence detection results showed that the mimics were efficiently transfected into 293 T cells (Figure 6A). The binding sites of miRNAs on the 3’UTR of candidate target genes are shown in Figure 6B. The luciferase assay results revealed that the relative luciferase activity was significantly lower in WT + mimics groups than Mut + mimics, psiCHECH2 + mimics, and psiCHECH2 + NC groups (*p* < 0.01), indicating that the mimics of miR-433-3p, miR-485-3p, miR-409-3p, and miR-495-3p could efficiently bind to their target sites and suppress the expression of luciferase (Figure 6C).

**Figure 7:**
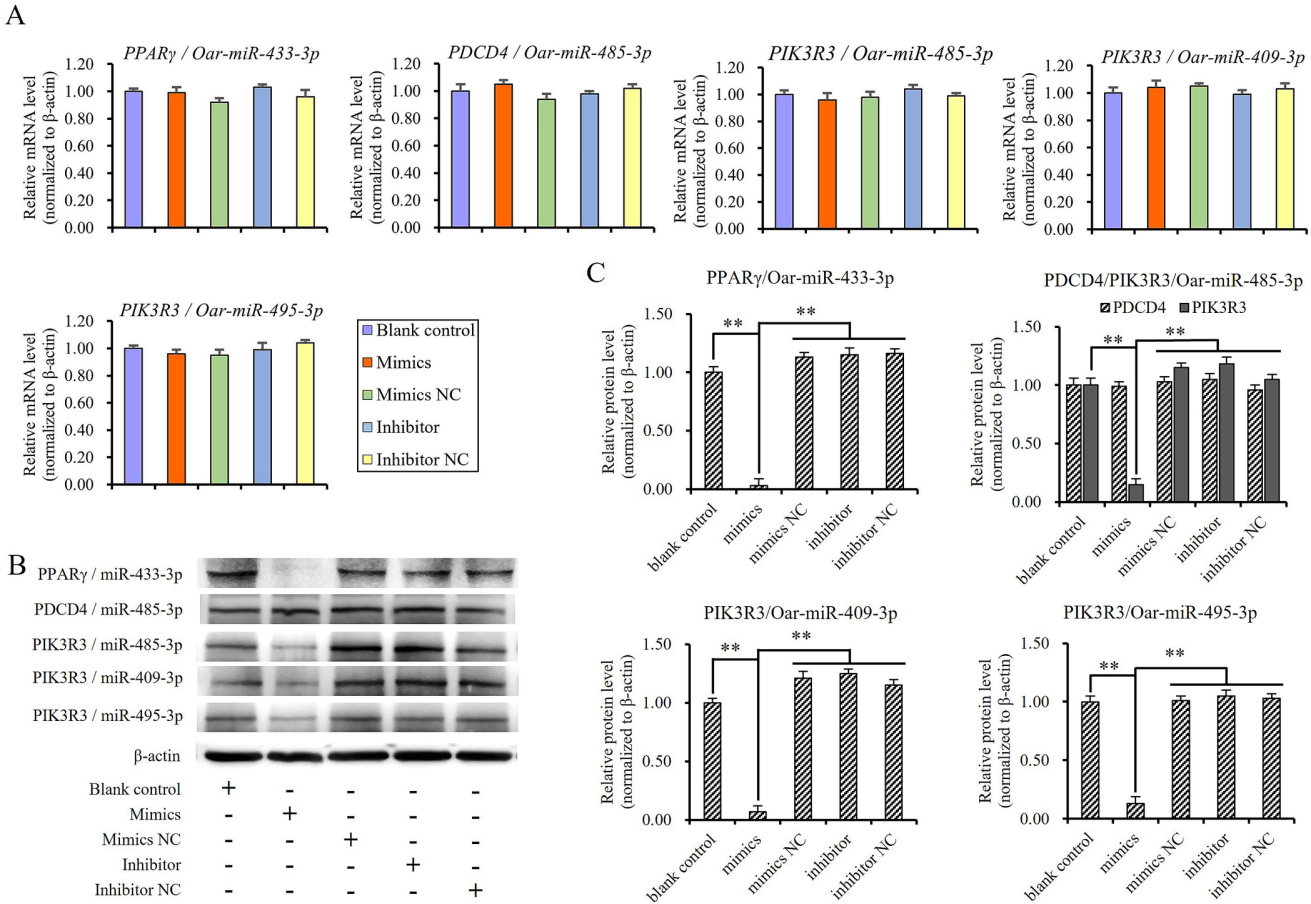
Validating the targeted regulatory relationship between miRNAs and candidate its target gene in sheep preadipocyte, **(A)** mRNA levels of target genes, **(B)** and **(C)** protein levels of target genes, **indicates significant difference (*p* < 0.01).

Then, sheep preadipocytes were transfected with mimics, mimics negative control, inhibitors, and inhibitors negative control of miR-433-3p, miR-485-3p, miR-409-3p, and miR-495-3p. The mRNA and protein levels of candidate target genes such as *PPARγ*, *PDCD4*, and *PIK3R3* were detected using qRT-PCR and Western blot after the cells were further cultured for 48 and 72 h. The results revealed no significant differences in the mRNA levels of *PPARγ*, *PDCD4*, and *PIK3R3* among the blank control group or any treatment groups involving these miRNAs (*p* > 0.05; Figure 7A). However, the protein levels of *PPARγ* and *PIK3R3* were significantly downregulated when mimics were transfected into cells comparing to other treatment groups (*p* < 0.01; Figures 7B,C). The results indicated that miR-433-3p, miR-485-3p, miR-409-3p, and miR-495-3p could suppress the transcription of mRNA of *PPARγ* and *PIK3R3*, but did not induce mRNA degradation because the mRNA levels did not show significant downregulation (*p* > 0.05). In terms of inhibitor groups, it was observed that although the single endogenous miRNA combined with its inhibitor failed to suppress the expression of its target gene, but there was no significant up-regulation in the protein levels of *PPARγ* and *PIK3R3* (*p* > 0.05; Figure 7B). This suggests that *PPARγ* and *PIK3R3* might be targeted by other multiple miRNAs.

It is evident that miR-485-3p did not suppress the expression of *PDCD4* in sheep preadipocytes (Figures 7B,C), although it has target site on the 3’UTR fragment of *PDCD4* (Figure 6B), and could effectively bind to it to suppress the expression of luciferase in dual-luciferase assay (Figure 6C). Thus, the luciferase assay may have a certain rate of false negatives, and the targeted regulatory relationship between miRNA and its candidate target gene should be finally validated by Western blot.

### 3.6 Influence of miRNA expression on the proliferation of preadipocyte

**Figure 8:**
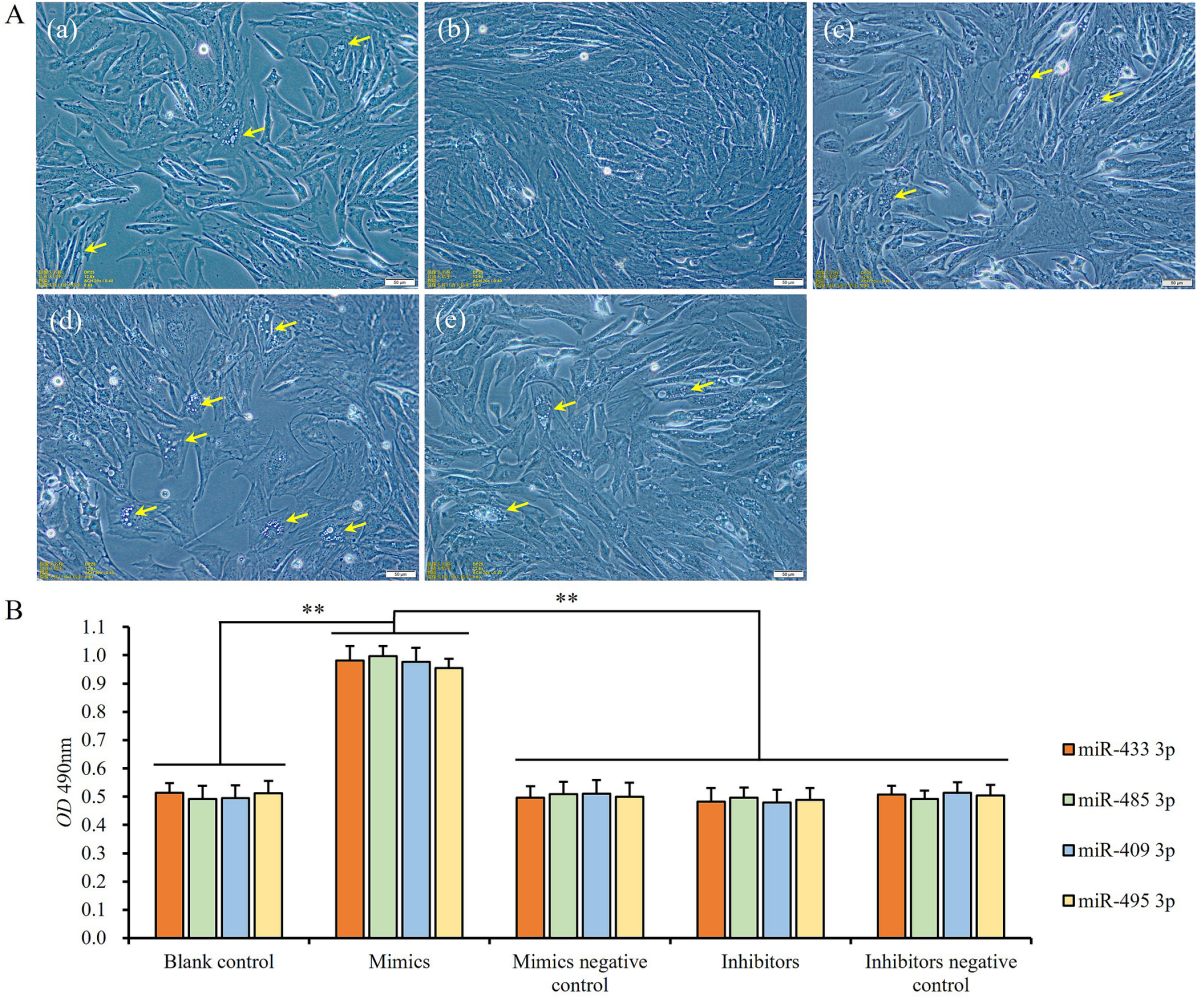
The influence of miRNA expression levels on proliferation and differentiation of preadipocytes: **(A)** cell states of blank control (a), miR-433-3p mimics (b), miR-433-3p negative control (c), miR-433-3p inhibitors (d), and miR-433-3p inhibitors negative control (e); the yellow arrows show the tiny lipid droplets; the scale line indicates 50 μm; **(B)** the difference of cell number among different groups; **indicates significant difference (*p* < 0.01).

When the expression levels of miRNAs were upregulated by transfecting mimics of miR-433-3p, miR-485-3p, miR-409-3p, and miR-495-3p, respectively, the preadipocytes exhibited a relatively rapid proliferation until reaching 100% confluent, and the adipogenic differentiation of cells was almost completely inhibited (Figures 8Ab,B). On the contrary, the cells in the blank control and rest treatment groups displayed slower proliferation than the mimics groups and gradually shifted to adipogenic differentiation with numerous tiny lipid droplets appearing within the cells (Figures 8Aa–e). Additionally, the number of cells was also significantly lower than that of mimics groups (*p* < 0.01; Figure 8B). In contrast, the inhibitors groups showed stronger adipogenic differentiation ability (Figure 8Ad) and slower rate of cell proliferation than the blank control, mimics negative control, inhibitors, and inhibitors negative control groups, but there was no significant difference among themselves (*p* > 0.05; Figure 8B).

### 3.7 Influence of miRNA expression on induced adipogenic differentiation of preadipocyte

**Figure 9:**
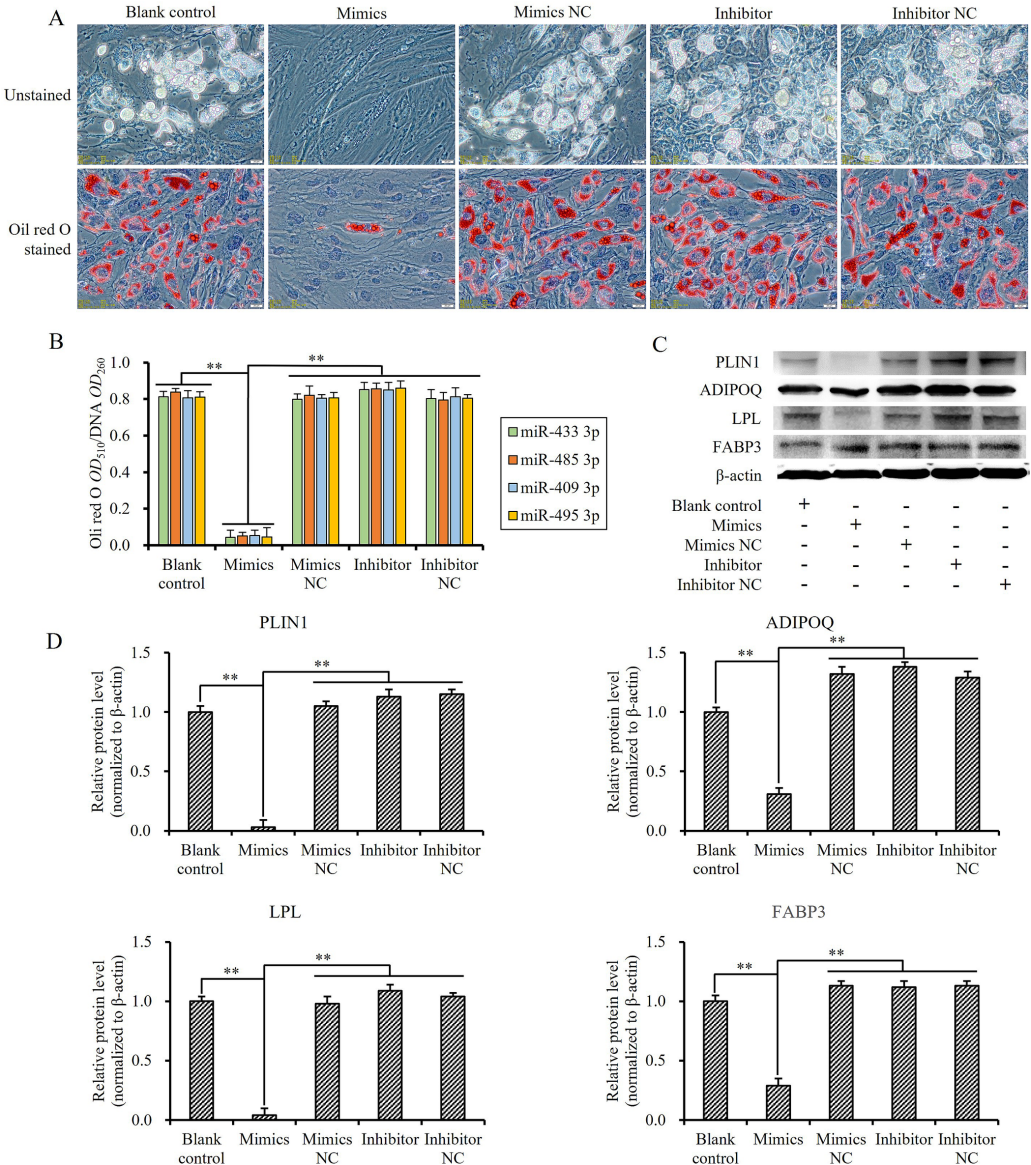
Influence of miRNA expression levels on lipogenesis-induced differentiation and gene expression of sheep preadipocytes related to fat metabolism: **(A)** differentiation state of sheep preadipocytes with different miR-433-3p expression levels, the scale line indicates 20 μm; **(B)** the amount of fat deposition in cells with different miRNA expression levels; **(C,D)** fluctuation of different miR-433-3p expression levels on expression of some key fat metabolism-related genes; **indicates a significant difference (*p* < 0.01).

When sheep preadipocytes, transfected with mimics, inhibitors, and NC of miR-433-3p, miR-485-3p, miR-409-3p, and miR-495-3p, respectively, were induced to undergo adipogenic differentiation, the cells in blank control, mimics negative control, inhibitors, and inhibitors negative control groups shifted to adipogenic differentiation states quickly. The tiny lipid droplets rapidly accumulated and then fused together into larger lipid droplets, but the adipogenic differentiation of the cells transfected with mimics was almost inhibited and kept stasis (Figure 9A). Moreover, these cells exhibited significantly lower levels of lipid deposition than those in the blank control group and other treatment groups (*p* < 0.01; Figure 9B).

The expression levels of four lipid metabolism-related genes, *FABP3*, *PLIN1*, *ADIPOQ*, and *LPL*, were detected to further investigate the effect of up- or downregulation of these four miRNAs. Following the transfection of miRNA mimics into sheep preadipocytes, the protein levels of FABP3, PLIN1, ADIPOQ, and LPL were significantly downregulated compared to both the blank control and other treatment groups (*p* < 0.01; Figures 9C,D). These data indicated that miR-433-3p, miR-485-3p, miR-409-3p, and miR-495-3p could inhibit the expression of their target genes *PPARγ* and *PIK3R3*, then regulate the expression of key genes related to lipid metabolism, finally inhibit the adipogenic differentiation of cells, and promote their proliferation *in vitro*.

## 4 Discussion

Just like the hump of a camel, the fat tail of sheep is an adaptive trait that facilitates their adaptation to the harsh natural environment in the temperate and cold regions on Earth during long and cold winters (8, 13). Sheep tail fat is subcutaneous adipose tissue, which grows through two ways, hypertrophy (increase in adipocyte volume due to deposition of lipids) and hyperplasia (increase in the number of adipocytes through proliferation and differentiation of precursors). The hyperplasia of preadipocytes occurs mainly in the fetal stage and the hypertrophy of adipocytes mainly after birth (25). Adipose tissues are typically surrounded by a fluid-filled network of collagen and elastin, with progenitor cells residing in the reticular interstitium. During summer and autumn when the grass is abundant, sheep obtain sufficient nutrition, and the proliferation of adipocyte progenitor cells is activated. While some cells still reside in the network, others enter the adipose tissue and differentiate into mature adipocytes and the number of adipocytes in tissue increases (26). Mature adipocytes store excess energy as lipids within cells, resulting in a noticeable increase in cell size. Hence, at the end of autumn, fat-tailed sheep typically exhibit significantly enlarged tail sizes (Figure 1A). In the long and cold winter, these fat-tailed sheep could obtain energy by decomposing tail fat to ensure their energy supply (27).

Several studies have detected the DE miRNAs in tail fat tissues of fat-tailed and thin-tailed sheep as well as at different development stages of fat-tailed sheep (16–18). MiRNA-Seq data of short-fat-tailed Hu sheep and short-thin-tailed Tibetan sheep revealed a total of 155 DE miRNAs, including 78 upregulated and 77 downregulated ones. Among them, 17 miRNAs were found to be involved in lipid metabolism (17). In the tail fat tissues of fat-tailed Sunite sheep aged 6, 18, and 30 months, a total of 219 DE miRNAs were detected, including 12 novel miRNAs (18). Meanwhile, 40 DE miRNAs were identified in the tail fat tissue of Guangling large-tailed sheep and small-tailed Han sheep (16). Compared to Hu, Tibetan, Sunite, and Guangling large-tailed sheep involved in the above investigation, the Altay sheep in the present research have a stronger tail fat deposition capacity and more significant tail fat traits (Figure 1). We speculated that the DE miRNAs in the tail fat tissues of Altay sheep at different developmental stages might be more significant. The miRNA-Seq data confirmed our hypothesis, a large number of DE miRNAs were identified in tail fat tissues at different developmental stages. In particular, miR-433, miR-485, miR-409, and miR-495 exhibited substantial upregulation of downregulation in one or more stages. Furthermore, the functional analysis demonstrated the close association of these DE miRNAs with signaling pathways involved in lipid metabolism such as the PPAR signaling pathway (ko03320), AMPK signaling pathway (ko04152), insulin signaling pathway (ko04910), mTOR signaling pathway (ko04150), and PI3K-AKT signaling pathway (ko04151). Therefore, further investigation is warranted to elucidate their roles in sheep tail fat deposition.

For the past few years, an increasing number of miRNAs and their target genes in fat deposition have been elucidated. The expression level of oar-miR-432 was significantly downregulated in the tail fat tissues of Tibetan thin-tailed sheep compared to Hu fat-tailed sheep. Further investigation revealed that oar-miR-432 could inhibit adipocyte differentiation by promoting the expression of bone morphogenetic protein 2 (*BMP2*) and inhibiting *PPARγ in vitro* (28). MiR-193a-5p showed significant differential expression in intramuscular fat of 2-month-old and 12-month-old Aohan fine-wool sheep, and it could inhibit 3 T3-L1 cells proliferation and differentiation by targeting acetyl-coenzyme A acyltransferase 2 (*ACAA2*) gene *in vitro* (29). MiR-27b could impair human adipocyte differentiation by targeting *PPARγ* (30). In human adipose tissue-derived stem cells (ASCs), miR-29b-3p upregulation contributed to ASCs differentiation into endothelial cells by targeting fibroblast growth factor receptor 1 (*FGFR1*), neuropilin 2 (*NRP2*), mitogen-activated protein kinase 1 (*MAPK1*), and transforming growth factor beta (*TGF-β2*) (31). Up to now, the roles of miR-433-3p, miR-485-3p, miR-409-3p, and miR-495-3p in fat deposition have not already been reported. In the present investigation, the high-throughput sequencing detected that these four miRNAs were expressed at a high level in the fetal stage but were significantly downregulated after birth. As preadipocytes proliferate mainly at the fetal stage (25) and the function analysis showed that their candidate target genes were involved in pathways related to lipid metabolism, we speculated that they might function by promoting preadipocyte proliferation and inhibiting differentiation. The results demonstrated that upregulation of these four miRNAs in sheep preadipocytes led to a significant downregulation of their target genes, *PPARγ* and *PIK3R3*, as well as several fat deposition-related genes, including *FABP3*, *PLIN1*, *ADIPOQ* and *LPL*. Additionally, cell proliferation was promoted while differentiation was inhibited (Figures 8, 9). *PPARγ* is an important transcription factor involved in the development and function of fat tissue, and genome-wide studies indicate that *PPARγ* binding sites are associated with a majority of genes activated during adipogenesis (32–34). As observed in the present study, the expression of downstream genes and adipogenesis were directly affected when the expression of *PPARγ* was inhibited by miR-433-3p.

Compared to *PPARγ*, *PIK3R3* may play a regulatory role at a higher level through the PI3K/AKT pathway, which has been proven as a positive regulator of adipocyte terminal differentiation (35, 36). In adipocytes, the PI3K/AKT pathway ultimately promotes the expression of *PPARγ* and CCAAT/enhancer-binding proteins (*C/EBPs*) by targeting forkhead box protein O1 (*FOXO1*) and accelerates the differentiation and maturation of preadipocytes (37). As the central kinase in the PI3K/AKT pathway, when *PIK3R3* expression was inhibited by miR-485-3p, miR-409-3p, and miR-495-3p, the expression of *PPARγ* was also inhibited simultaneously. Therefore, we speculated that, in tail fat tissues, the expression of *PPARγ* was regulated by at least two factors, miR-433-3p and PIK3R3.

Based on the present results, we can draw an outline of the regulatory approach of these four miRNAs in sheep fat tail development. In the fetal period, these four miRNAs were expressed at a high level, so the expression of *PIK3R3* and *PPARγ* was inhibited, and the preadipocytes started proliferating to store enough cells for tail fat tissue growth after born, but their differentiation was inhibited. After the lamb was born, the expression of these four miRNAs was downregulated, so the inhibition of *PIK3R3* and *PPARγ* was removed. The preadipocytes start to differentiate and become mature adipocytes by depositing lipids in cells, and tail fat tissues gradually grow by hypertrophy of adipocytes. Of course, partial preadipocytes may start proliferate again to supply the number of adipocytes in the fat tail (38, 39). Meanwhile, the mature adipocytes could also dedifferentiate into preadipocytes and then differentiate into adipocytes again when the nutrition is sufficient (40–42).

## 5 Conclusion

In the present study, we investigated the expression profile of miRNAs induced during fat tail development of fat-tailed Altay sheep, which have strong tail fat deposition ability, and thin-tailed XFW sheep, which have relatively poor tail fat deposition ability. Plenty of DE miRNAs were identified, and subsequent GO and KEGG analysis revealed that these DE miRNAs and their candidate target genes were involved in several important signaling pathways related to adipogenesis. Further investigation proved that four key DE miRNAs, miR-433-3p, miR-485-3p, miR-409-3p, and miR-495-3p, could promote the proliferation of sheep adipocytes and inhibit their differentiation by targeting *PPARγ* and *PIK3R3*.

## Data Availability

The datasets presented in this study can be found in online repositories. The names of the repository/repositories and accession number(s) can be found in the article/Supplementary material.

## Footnotes

^1^ http://www.mirbase.org

^2^ https://www.ebi.ac.uk/ena/browser/view/GCA_000298735.1

^3^ www.targetscan.org

^4^ www.microRNA.org

^5^ http://www.geneontology.org

^6^ http://www.genome.jp

^7^ https://hiplot.com.cn/

## Supplementary material

Supplementary Table S1 Primers used in present study.

Supplementary Table S2 Distribution of small RNA among different categories.

Supplementary Table S3 Differentially expressed miRNAs analysis.

Supplementary Table S4 Candidate target genes of top 20 DE miRNAs.

Supplementary Table S5 GO and KEGG analysis of target genes of top 20 DE miRNAs.
